# Survival Outcomes of Nonsmall Cell Lung Cancer Patients Treated with Afatinib Who Are Affected by Early Adverse Events

**DOI:** 10.1155/2021/2414897

**Published:** 2021-06-16

**Authors:** Jessica M. Logan, Doug A. Brooks, Andrew Rowland, Michael J. Sorich, Ashley M. Hopkins

**Affiliations:** ^1^Clinical and Health Sciences, Cancer Research Institute, University of South Australia, North Terrace, Adelaide 5001, South Australia, Australia; ^2^College of Medicine and Public Health, Flinders University, Flinders Drive, Bedford Park, Adelaide 5042, South Australia, Australia

## Abstract

**Introduction:**

Afatinib is a first-line treatment option for patients with an advanced nonsmall cell lung cancer (NSCLC) expressing an epidermal growth factor receptor (EGFR) activating mutation. This study aimed to evaluate the association between early adverse events induced by afatinib and overall survival (OS) and progression free survival (PFS) in patients with advanced NSCLC.

**Methods:**

The study was a pooled post hoc analysis of the randomized trials LUX-Lung 3 and LUX-Lung 6 which evaluated afatinib versus pemetrexed-cisplatin or gemcitabine-cisplatin, respectively. Cox proportional hazard analysis was used to assess the impact of adverse events occurring within the first 28 days of afatinib therapy on the PFS and OS outcomes in treatment-naïve advanced NSCLC patients harbouring an EGFR activating mutation.

**Results:**

There were 468 patients who initiated first-line afatinib therapy within LUX-Lung 3 and LUX-Lung 6. A significant association between early rash and improved OS (hazard ratio (HR 95% CI); grade 1 = 0.74 [0.56–0.97]; grade 2+ = 0.64 [0.46–0.89]) (*P* = 0.018) was observed, although no significant association with PFS was present (*P* = 0.732). A significant association was identified between early diarrhoea and improved PFS (grade 1 = 0.83 [0.62–1.12]; grade 2+ = 0.62 [0.44–0.88]) (*P* = 0. 015), although no significant association with OS was present (*P* = 0.605). No associations between early stomatitis or paronychia and OS or PFS were identified.

**Conclusion:**

Rash occurring early after the initiation of afatinib was significantly associated with improved OS, an indicator that rash may be a surrogate of patients likely to achieve long-term survival. Consideration of using rash as a dose adjustment target may be warranted for future prospective trials aiming to optimise outcomes with afatinib therapy.

## 1. Introduction

The incidence of lung cancer in 2021 is predicted to be over 2.1 million cases with 1.8 million cancer-associated deaths, making it the leading cause of cancer death worldwide. The vast majority of lung cancers (>84%) are characterised as nonsmall cell lung cancer (NSCLC) and with 33.7% of these patients identified as having a mutation in the epidermal growth factor receptor (EGFR), which results in constitutive activation of tyrosine kinase activity [1–5]. First-line treatment options for NSCLC tumours expressing an EGFR activity enhancing mutation include the second-generation small molecule EGFR inhibitor, afatinib. Afatinib, as well as its first-generation EGFR inhibitor counterparts (erlotinib and gefitinib), is most active against tumours expressing EGFR exon 19 or L858R missense mutations [6–8]. However, unlike first-generation EGFR inhibitors, afatinib irreversibly binds to the EGFR receptor, which is thought to reduce resistance and relapse occurrence [9]. Afatinib treatment has been proven to improve survival outcomes, when compared to first-generation EGFR inhibitors, such as gefitinib [10]. The increased therapeutic efficacy of afatinib is associated with increased toxicity, although the rates of discontinuation are similar for the different EGFR inhibitors [10].

The adverse events most commonly reported with afatinib treatment include diarrhoea, acneiform rash, stomatitis/mucositis, and paronychia [6, 8, 11, 12]. Emerging evidence indicates that the occurrence of early adverse events following the initiation of targeted cancer therapies might be associated with superior overall survival (OS) or progression free survival (PFS) [13–16]. For example, the occurrence of early onset treatment arthralgia is associated with superior survival outcomes from vemurafenib plus cobimetinib treatment for melanoma, providing evidence for a potential dose-escalation marker to improve outcomes with this treatment combination [14]. Conversely, the occurrence of diarrhoea is associated with worse OS and PFS in metastatic colorectal cancer patients treated with ramucirumab and FOLFIRI chemotherapy, which necessitates the increased use of preventative antidiarrheal medications in high risk patients [13]. To date, there has been minimal exploration of the association between early afatinib-induced adverse events and survival outcomes.

This study aimed to evaluate the association between early adverse events induced by afatinib treatment and OS and PFS in patients with advanced NSCLC.

## 2. Materials and Methods

### 2.1. Data

The study was a pooled post hoc analysis of individual-participant data (IPD) from the clinical trials LUX-Lung 3 (NCT00949650) [11, 12] and LUX-Lung 6 (NCT01121393) [8, 11, 12]. LUX-Lung 3 and LUX-Lung 6 were randomized trials of afatinib versus pemetrexed-cisplatin or gemcitabine-cisplatin, respectively, for treatment-naïve advanced NSCLC harbouring an EGFR activating mutation [8, 11, 12]. The analysed data included the per-protocol patients from the afatinib arms of LUX-Lung 3 and LUX-Lung 6. Secondary analysis of anonymized clinical trial data was confirmed as negligible risk research by the Southern Adelaide Local Health Network, Office for Research and Ethics, and was exempt from review.

## 3. Outcomes and Predictors

OS was the primary outcome, and PFS was assessed as a secondary outcome. OS was defined from the date of the first dose of afatinib (randomization) to the date of the last follow-up or death. PFS was defined from the date of the first dose of afatinib (randomization) to the date of disease progression or death, whichever occurred first. Disease progression was assessed by the investigators according to the Response Evaluation Criteria in Solid Tumours (RECIST): version 1.1 for LUX-Lung 3 and 6.

Adverse events and their severity were defined by grade according to the National Cancer Institute Common Terminology Criteria for Adverse Events v3.0. Specific adverse events that were common early after afatinib initiation were evaluated for association with OS and PFS, which included stomatitis, paronychia, rash, and diarrhoea.

### 3.1. Statistical Analysis

The associations between early adverse events and OS/PFS for participants initiated on afatinib were assessed using a landmark Cox proportional hazard approach. The landmark was set at 28 days following afatinib initiation; with individuals who progressed or died within the first 28 days being excluded. Such an approach is required to minimise the potential for immortal time bias/guarantee time bias that are introduced if participants who have died or progressed before the landmark time are included in the analysis [17–19]. The methodologies were adapted from prior published work [13, 14, 16–19]. The 28-day landmark point was derived according to a balance between being as early as possible (as early markers are more clinically useful, and study power decreases as the more patients progress or die) and ensuring enough adverse events had occurred before the landmark time. Complete case analyses were conducted, and associations were reported as hazard ratios (HR) with 95% confidence intervals (95% CI) and *P* values (likelihood ratio test). All models were stratified by the study. For significant univariables, analyses adjusted for pretreatment age, sex, race, Eastern Cooperative Oncology Group performance status (ECOG PS), smoking status, stage, and EGFR mutation type were conducted. Kaplan–Meier analysis was used to plot OS and PFS estimates for identified associations. All statistical analyses were performed with R (version 3.4.3).

## 4. Results

### 4.1. Patient Population

Within the available data, 468 patients initiated first-line afatinib therapy (Supplementary Table S1 provides a summary of patient characteristics). Fourteen participants were excluded from the analysis due to disease progression, death, or loss to follow-up before day 28. Stomatitis, paronychia, rash, and diarrhoea were the most frequent adverse events following afatinib initiation (Supplementary Table S2).

### 4.2. Association between Early Adverse Events and Survival Outcomes

**Figure 1:**
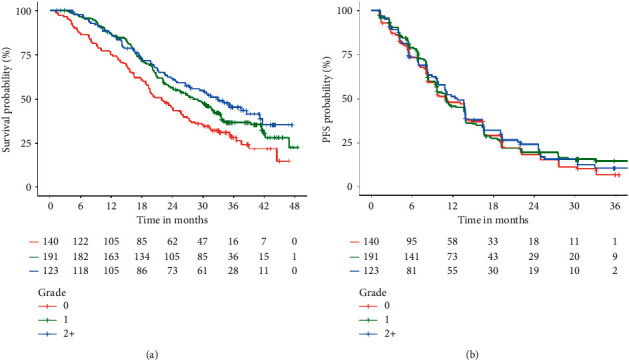
Kaplan–Meier estimates of OS and PFS by the maximum grade of rash within the first 28 days of afatinib treatment.

**Figure 2:**
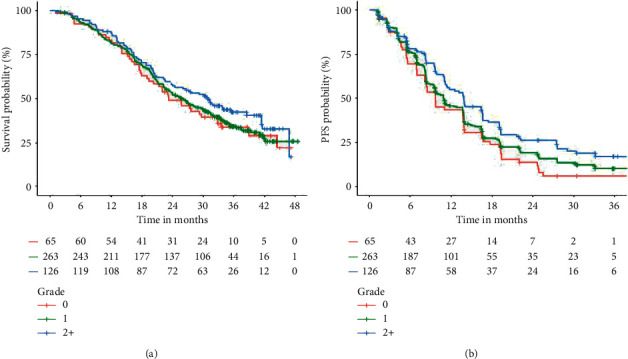
Kaplan–Meier estimates of OS and PFS by the maximum grade of diarrhoea within the first 28 days of afatinib treatment.

Table 1 summarizes the associations between specific adverse events occurring within the first 28 days of afatinib therapy with OS and PFS. A significant association between rash within the first 28 days of afatinib therapy and improved OS (HR [95% CI]; grade 1 = 0.74 [0.56–0.97]; grade 2+ = 0.64 [0.46–0.89]; *P* = 0.018) was observed, but there was no statistically significant association with PFS (HR [95% CI]; Grade 1 = 0.91 [0.71–1.18]; grade 2+ = 0.90 [0.68–1.21]; *P* = 0.732) (Figure 1). There was a significant association between diarrhoea within the first 28 days of afatinib therapy and improved PFS (HR [95% CI]; grade 1 = 0.83 [0.62–1.12]; Grade 2+ = 0.62 [0.44–0.88]; *P* = 0.015), but no statistically significant association with OS (HR [95% CI]; Grade 1 = 0.95 [0.68–1.33]; grade 2+ = 0.84 [0.58–1.23]; *P* = 0.605) (Figure 2). There were no associations between stomatitis and paronychia within the first 28 days of afatinib therapy and OS or PFS.

On adjusted analysis, there was also a significant association between rash and improved OS (HR [95% CI]; grade 1 = 0.74 [0.55–0.99]; grade 2+ = 0.60 [0.43–0.85]; *P* = 0.012), but not PFS (HR [95% CI]; grade 1 = 0.93 [0.71–1.22]; grade 2+ = 0.86 [0.63–1.18]; *P* = 0.647). Similarly, the association between diarrhoea and PFS (HR [95% CI]; grade 1 = 0.81 [0.59–1.10]; grade 2+ = 0.60 [0.42–0.85]; *P* = 0.012) was significant, but not for OS (HR [95% CI]; grade 1 = 0.87 [0.62–1.23]; grade 2+ = 0.68 [0.46–1.01]; *P* = 0.117).

## 5. Discussion

In a large high-quality dataset, this study for the first time investigated the association between early adverse events induced by afatinib and survival outcomes in advanced NSCLC. Afatinib-induced rash was identified as significantly and independently associated with improved OS, while diarrhoea was associated with improved PFS.

Afatinib is a second-generation EGRF inhibitor, which irreversibly binds to the tyrosine kinase domain of the epithelial growth factor receptor (EGFR) to block downstream signalling and growth pathways in solid tumours [6–8]. Afatinib, as well as its first-generation counterparts, targets the conserved tyrosine kinase domain, with additional targets including EGFR (ErB1), HER2 (ErB2), and HER4 (ErB4) [6, 7]. Ordinarily, the EGFR pathway stimulates tumour cell proliferation, migration, adhesion, and angiogenesis and inhibits apoptosis. Overexpression of EFGR has been associated with increased metastasis, reduced survival, and poorer prognosis [20]. EGRF is also highly expressed in normal epithelial cells and thus the occurrence of cutaneous reactions with afatinib is linked to the biological mechanisms combating cancer. Thus, it is not surprising that in the current study, rash was identified as a surrogate marker for improved OS with afatinib treatment [20–22]. These results are also similar to prior studies reporting improved OS and PFS in advanced cancer patients treated with erlotinib and gefitinib who experience rash [23–26]. Like the epidermis, the lining of the colon is epithelium based. Thus, like the identified rash-OS association, it is not surprising that a gastrointestinal-related adverse event such as diarrhoea may similarly be linked to improved survival outcomes with afatinib treatment.

While afatinib-induced rash was associated with improved OS, no statistically significant association with PFS was identified. Vice versa, afatinib-induced diarrhoea was associated with improved PFS, but not OS. The reasons for this discrepancy are unclear; however, PFS is an imperfect surrogate marker, which may be affected by variability in timing of assessments, investigators, and measurement biases [27, 28]. Opposing this, OS is confounded by drug cessation, crossover, and subsequent therapies [27, 28]. An appreciation of these points indicates that the rash associations may not be isolated to afatinib treatment and rather represents patients with a greater sensitivity to many anticancer medicines which can compound across multiple treatments or is an indicator of a more functional immune system. For example, rash has been associated with improved survival outcomes for many anticancer medicines, including chemotherapies [29–31]. However, diarrhoea and improved outcomes may be afatinib specific; for example, diarrhoea has been associated with worsened survival outcomes for other anticancer treatment options [13, 32–34].

Importantly, the present study suggests that early rash could function as a clinical surrogate of patients likely to achieve long-term survival benefit from a tailored dose of afatinib. As demonstrated here, this association is also likely to be dependent upon the pharmacodynamic pathway of afatinib and the overexpression of EGFR in cancer and epithelial cells [6]. Interestingly, future research may consider using rash as a dose-escalation marker to improve long-term clinical outcomes in advanced NSCLC patients treated with afatinib. Such an intervention has previously been identified as a successful strategy for other anticancer medicines [14, 30]. Key to implementing toxicity-based dosing strategies is small incremental dose increases till the occurrence of a manageable grade 1 or 2 rash, which acts as a surrogate that enough drug is at the target cancer site. Of caution is that toxicity of grade 3 or above may result in the need for dose interruptions or reductions, which ultimately may negatively impact on survival outcomes [14].

The large high-quality dataset used in this post hoc analysis was resultant from pooling two clinical trials. A significant strength of the study is that the dosing protocols of LUX-Lung 3 and LUX-Lung 6 are consistent with contemporary afatinib administration. Furthermore, the study used a landmark approach to minimise the potential for immortal time bias, and the 28-day timeframe is early enough to be actionable in clinical practice. A potential limitation of the study is that clinical trials do not include all of the patients that may be treated within clinical practice. For example, LUX-Lung 3 and LUX-Lung 6 included only patients with Eastern Cooperative Oncology Group (ECOG) performance status of 0 or 1 [8, 11, 12], which may limit the generalizability of the findings. Future studies will have a role in investigating the identified associations in real-world cohorts and the LUX-Lung 7 trial presents another cohort of interest.

In conclusion, in a large high-quality data, the onset of rash was associated with improved OS and diarrhoea with improved PFS in advanced NSCLC patients treated with afatinib. This suggests that early rash could function as a surrogate marker of long-term survival benefit. Future research may consider the exploration of dose-escalation to grade 1 or 2 rash as a mechanism to improve long-term clinical outcomes in advanced NSCLC patients treated with afatinib.

## Figures and Tables

**Table 1:** Summary of the association between adverse events within the first 28 days of afatinib treatment and OS or PFS.

*N*		Overall survival			Progression free survival		
		Median (months)	HR [95% CI]	*P* value	Median (months)	HR [95% CI]	*P* value
*Stomatitis*				0.631			0.476
0	222	27	1.00		11	1.00
1	165	25	1.12 [0.86–1.46]		11	0.95 [0.75–1.20]
2+	67	27	1.13 [0.80–1.60]		14	0.81 [0.58–1.14]

*Rash*				0.018			0.732
0	140	22	1.00		11	1.00
1	191	28	0.74 [0.56–0.97]		11	0.91 [0.71–1.18]
2+	123	33	0.64 [0.46–0.89]		13	0.90 [0.68–1.21]

*Paronychia*				0.232			0.112
0	396	26	1.00		11	1.00
1	36	31	0.84 [0.54–1.30]		11	0.85 [0.57–1.26]	
2+	22	42	0.63 [0.34–1.17]		15	0.61 [0.36–1.03]	

*Diarrhoea*				0.605			0.015
0	65	23	1.00		10	1.00
1	263	26	0.95 [0.68–1.33]		11	0.83 [0.62–1.12]
2+	126	31	0.84 [0.58–1.23]		14	0.62 [0.44–0.88]

HR = hazard ratio; CI = confidence interval.

## Data Availability

Individual-participant data utilised were accessed via clinicalstudydatarequest.com according to the Boehringer Ingelheim policy and process for clinical study data sharing.

## Supplementary Materials

Supplementary Materials Supplementary Tables S1 and S2 provide summaries of patient characteristics and maximum grade of adverse events within the first 28 days of afatinib administration.
