# Supplementary material for: Survival Outcomes of Nonsmall Cell Lung Cancer Patients Treated with Afatinib Who Are Affected by Early Adverse Events
Source: J Oncol. 2021 Jun 16;2021:2414897. doi: 10.1155/2021/2414897 (PMC8225415; doi:10.1155/2021/2414897)
Supplement: Supplementary Materials — Supplementary Tables S1 and S2 provide summaries of patient characteristics and maximum grade of adverse events within the first 28 days of afatinib administration. [file 2414897.f1.docx]

# Supplementary results

Supplementary Table 1: Summary of patient characteristics by study

|  | Total No. 468 | LUX-Lung 3 No. 229 | LUX-Lung 6 No. 239 | P-value |
| --- | --- | --- | --- | --- |
| Age (years) | 60 (51 - 67) | 62 (55 - 68) | 58 (49 - 65) | < 0.001 |
| Sex | | | | 0.92 |
| Male | 169 (36%) | 82 (36%) | 87 (36%) |  |
| Female | 299 (64%) | 147 (64%) | 152 (64%) |  |
| Race | | | | < 0.001 |
| American Indian or Alaska Native | 2 (0%) | 2 (1%) | 0 (0%) |  |
| Asian | 404 (86%) | 165 (72%) | 239 (100%) |  |
| Black | 1 (0%) | 1 (0%) | 0 (0%) |  |
| White | 61 (13%) | 61 (27%) | 0 (0%) |  |
| ECOG PS | | | | < 0.001 |
| 0 | 140 (30%) | 92 (40%) | 48 (20%) |  |
| 1 | 328 (70%) | 137 (60%) | 191 (80%) |  |
| Smoking status | | | | < 0.001 |
| Current | 22 (5%) | 5 (2%) | 17 (7%) |  |
| Former | 114 (24%) | 70 (31%) | 44 (18%) |  |
| Never | 332 (71%) | 154 (67%) | 178 (74%) |  |
| Stage | | | | 0.29 |
| IIIB | 34 (7%) | 20 (9%) | 14 (6%) |  |
| IV | 434 (93%) | 209 (91%) | 225 (94%) |  |
| EGFR mutation | | | | 0.77 |
| Del19 | 235 (50%) | 111 (48%) | 124 (52%) |  |
| L858R | 180 (38%) | 91 (40%) | 89 (37%) |  |
| Other | 53 (11%) | 27 (12%) | 26 (11%) |  |
| Data are median (IQR) or number of patients (%). | | | | |

Supplementary Table 2: Summary of maximum grade of adverse event within the first 28 days of afatinib administration.

| Grade | Total No. 468 | LUX-Lung 3 No. 229 | LUX-Lung 6 No. 239 | P-value |
| --- | --- | --- | --- | --- |
| Stomatitis | | | | < 0.001 |
| 0 | 230 (49%) | 85 (37%) | 145 (61%) |  |
| 1 | 167 (36%) | 98 (43%) | 69 (29%) |  |
| 2+ | 71 (15%) | 46 (20%) | 25 (10%) |  |
| Paronychia | | | | 0.001 |
| 0 | 410 (88%) | 188 (82%) | 222 (93%) |  |
| 1 | 36 (8%) | 24 (10%) | 12 (5%) |  |
| 2+ | 22 (5%) | 17 (7%) | 5 (2%) |  |
| Rash | | | | < 0.001 |
| 0 | 150 (32%) | 47 (21%) | 103 (43%) |  |
| 1 | 194 (41%) | 102 (45%) | 92 (38%) |  |
| 2+ | 124 (26%) | 80 (35%) | 44 (18%) |  |
| Diarrhoea | | | | 0.001 |
| 0 | 69 (15%) | 27 (12%) | 42 (18%) |  |
| 1 | 267 (57%) | 120 (52%) | 147 (62%) |  |
| 2+ | 132 (28%) | 82 (36%) | 50 (21%) |  |
| Data are median (IQR) or number of patients (%). | | | | |
